# Scoping Review: Physical Activity and Social Functioning in Young People With Autism Spectrum Disorder

**DOI:** 10.3389/fpsyg.2019.00120

**Published:** 2019-02-13

**Authors:** Nicole J. Reinders, Alexandra Branco, Kristen Wright, Paula C. Fletcher, Pamela J. Bryden

**Affiliations:** Department of Kinesiology, Wilfrid Laurier University, Waterloo, ON, Canada

**Keywords:** autism, physical activity, social functioning, review, intervention

## Abstract

Autism Spectrum Disorder (ASD) affects ~1 in 59 people in North America and diagnoses continue to rise (Center for Disease Control and Prevention, 2018). Unfortunately, the exact cause of ASD is unknown and therapy remains the primary means of intervention. People with ASD experience social and behavioral deficits associated with the disorder, which affect all aspects of life such as academics, relationships, and physical activity. Research has shown a relationship between physical activity and social skills in typically developing individuals; however, this relationship is less understood in people with ASD. The purpose of this scoping review was to uncover what is known about ASD, physical activity, and social functioning. The authors searched four databases and included 40 primary research articles in the review, most of which demonstrated a relationship between physical activity and social functioning for people with ASD. The relationship appears bidirectional: social functioning influences physical activity (to a lesser extent) and physical activity influences social functioning (to a greater extent). Regrettably, there were many limitations in these articles, such as small sample sizes and the under-representation of females and adults. Therefore, the review highlights several directions for future research.

## Introduction

Autism Spectrum Disorder (ASD) affects an individual's language skills and ability to think, feel, and relate to others (American Psychiatric Association, 2013). Medical professionals use the Diagnostic and Statistical Manual of Mental Disorders (DSM-5) to diagnose ASD and assign severity, ranging from level 1 (requiring support) to level 3 (requiring very substantial support), markedly defining it as a spectrum disorder (Autism Canada, 2015). People with ASD are characterized by deficits in social skills, repetitive and restricted behaviors and interests (American Psychiatric Association, 2013). In North America, the prevalence of ASD is ~1 in 59, with 4 males diagnosed for each female (Center for Disease Control and Prevention, 2018).

ASD is characterized by social and behavioral deficits; however, children with ASD may also experience motor deficits as compared to typically developing (TD) children (Sparaci et al., 2015). Specifically, children with ASD aged 7 to 10 years scored lower on the Movement Assessment Battery for Children than TD children, primarily in complex tasks such as balance and catching (Whyatt and Craig, 2012). Further, motor skills have been shown to predict social communication deficits (*p* < 0.05) in children with ASD (MacDonald et al., 2013). Casartelli et al. (2016) contended an association between the motor anomalies present in children with ASD and the social deficits characteristic of the disorder. The authors suggest that motor cognition–the ability for someone to understand phenomena through physical means – should be considered when diagnosing and subsequently treating ASD (Casartelli et al., 2016). Further, van der Fels et al. (2015) discovered associations between motor and cognitive skills, suggesting “complex motor intervention programs can be used to stimulate both motor and higher order cognitive skills in pre-pubertal children” (p. 697).

Based on this research, treatment addressing motor skills may positively influence the social functioning of people with ASD. In the review by Wong et al. (2014), exercise was found as an evidence-based practice to “address behavior, school-readiness, academic, and motor skills” (p. 58). Not only is physical activity (PA) a viable intervention to improve motor skills for people with ASD, it may also provide opportunities for social development. Pan et al. (2011) reported a positive correlation between social engagement and moderate to vigorous PA (MVPA) in adolescent males with ASD.

Peers and friends may positively and negatively influence PA among children and adolescents with ASD, regardless of social impairments (Obrusnikova and Cavalier, 2011). Social skills can promote positive peer relationships and personal competence, which in turn may promote the development of both social skills and movement behaviors (Pan et al., 2011). In addition, the review by Sowa and Meulenbroek (2012) found improved social and motor skills after exercise-based interventions in people with ASD. Conversely, in Pan's study (2009), PA levels were not dependent on social engagement. Based on this literature, it appears there may be interactions between social skills, peer relationships, and PA for people with ASD. Social, behavior, and motor impairments have been shown to contribute to sedentary behavior and obesity among young people with ASD (Srinivasan et al., 2014).

The current literature regarding the amount PA participation of people with ASD is inconclusive, but it appears those with ASD spend less time in moderate to vigorous PA and more time in sedentary behavior than their TD peers (Jones et al., 2017). Currently, an estimated 9% of Canadians aged 5 to 17 years meet the suggested 60 min of moderate to vigorous PA each day (Statistics Canada, 2017). These rates are concerning, as physical inactivity is linked to many preventable health conditions such as diabetes and depression, which cannot be addressed solely via nutrition and diet interventions (World Health Organization, 2018). Further, PA significantly affected social skills in TD children (Zurc, 2012) and leisure-time PA involvement has shown a dose-dependent relationship upon physical, social, and mental wellbeing in TD adults, both short term (at baseline) and long-term (4- and 8-year follow-ups) (Sanchez-Villegas et al., 2012).

Previous literature both supports and refutes the contention that people with ASD participate in an adequate amount of daily physical activity. For instance, children with ASD had similar PA levels as TD children based on accelerometer data; however, parent reports indicated that children with ASD had lower levels of PA and participated in a narrower range of activities than the TD children (Bandini et al., 2013). Further, Ketcheson et al. (2018) found that preschoolers with ASD were less sedentary than TD children, while Pan et al. (2011) found the opposite. However, there is encouraging evidence overall suggesting that people with ASD are at least capable of reaching the physical activity guidelines for TD individuals (Tyler et al., 2014).

According to the social model of disability, stigmas associated with impairments are rooted in social views of normalcy (Llewellyn and Hogan, 2000). This model may shed light on the PA behaviors of people with ASD due to their potential lack of control over and accessibility to the environment as compared to the general population (Pan and Frey, 2006). Access to recreational PA programming for young people with ASD may be affected by societal norms, thus resulting in inequitable opportunities for participation as compared to TD individuals. Consequently, the social model rationalizes that PA participation is more greatly affected by social barriers than by the specific symptoms of ASD (Llewellyn and Hogan, 2000). As a result, PA is not viewed as high-priority for young people with ASD, as there are more pressing issues such as overcoming difficulties with communication and reducing stereotypic behaviors.

In addition to varying PA levels, the current literature also shows conflicting results regarding the effects of PA for people with ASD. Lang et al. (2010) contend that PA reduced negative behaviors (e.g., stereotypy) and increased positive behaviors (e.g., academic engagement) in people aged 3 to 41 with ASD. Further, Yang et al. (2015) found increased motor performance and positive behaviors in young people with ASD as a result of PA, but the relationship between PA and cognition remained unclear. From a recent meta-analysis, fundamental motor skill training was positively correlated with PA and MVPA and negatively correlated with sedentary behavior in TD preschoolers (Engel et al., 2018), but the review by Ketcheson et al. (2018) revealed no relationship between PA and motor skills in preschoolers with ASD.

The Physical Activity Behavior model (Van der Ploeg et al., 2004) highlights the dependency of PA behaviors of people with disabilities upon personal factors (e.g., attitude and feelings of self-efficacy toward PA) and environmental factors (e.g., social influences from friends and family). This model is highly moderated by symptoms associated with a particular disability. For people with ASD, social deficits may act as barriers to PA, as PA often occurs in social environments (Obrusnikova and Miccinello, 2012). Common social symptoms include avoidance of eye contact, inability to play with others, and trouble understanding and expressing feelings (Center for Disease Control and Prevention, 2018). As such, engagement in recreational activities with peers (such as PA) may be difficult for people with ASD.

Many barriers and facilitators of PA have been identified through this model. For instance, environmental factors such as program availability, location, and cost, highly influence whether any individual will become active, regardless of ability level (Van der Ploeg et al., 2004). These barriers have been exacerbated for people with disabilities such as ASD. Socially, friends, family members, and health professionals of people may have poor attitudes toward PA or low expectations of PA, which negatively influences PA levels regardless of physical or financial accessibility to programs (Van der Ploeg et al., 2004).

Based on the literature reviewed above, it appears as though people with ASD may be engaged in more sedentary activities than TD individuals. Further, it appears the social and behavioral deficits associated with the disorder may be related to PA (e.g., are the result of reduced PA in development or cause reduced PA over the lifespan). Because ASD is a spectrum disorder, there may be differences for individuals based on the severity of their symptoms. Consequently, there seems to be a need to review literature regarding ASD, PA, and social functioning (SF) in more detail to: (1) identify areas for future research, and (2) develop strategies for caregivers and healthcare professionals who work with people with ASD. A scoping review was deemed most appropriate for these outcomes because it is exploratory in nature and incorporates a variety of research designs, focusing on breadth rather than depth. All methodologies will be considered in the process of this review to ensure breadth of study, as there are few studies which marry together PA and SF for people with ASD. Therefore, the scoping review will identify the feasibility of future work in this area from a variety of methodological perspectives. To date, no reviews have been conducted regarding this purpose. This paper will summarize the state of the current literature on PA and SF for people with ASD and identify gaps which will provide direction for future research in the area.

## Methods

The review was carried out following the framework outlined by Arksey and O'Malley (2005). For other reviews that have utilized this framework see Kushki et al. (2011); Edwards et al. (2017); and Williams and Reddy (2016). The five stages are outlined below.

### Stage 1: Identify the Research Question

The purpose of this review was to explore the relationship between PA and SF for people with ASD. This research question followed Arksey and O'Malley's (2005) suggestion to start with a broad review area to determine what is available before narrowing the search. The authors of the current review are researchers in the field of ASD and have reason to believe these relationships exist, both from personal experiences and published literature, but the existing research is contradictory. Identifying research questions was necessary for directing the review and determining how the relevant studies will be identified and selected. The research questions of this review are: (1) are PA and SF related? (2) does training one ability (e.g., PA or SF) have any effect on the other? and (3) what are the implications of this research?

### Stage 2: Identify Relevant Studies

#### Search Terms

Key terms were selected to locate studies pertinent to the research questions outlined. The search terms used were as follows: [“Physical Activity” OR Exercise OR Sport OR Recreation OR Fitness OR “Physical Education”] for PA, [Social OR Facilitator OR Barrier OR Peer OR Family OR Behavio^*^] for SF, and [Autis^*^ OR Asperger^*^ OR “Autism Spectrum Disorder”] for ASD. The search terms were entered into the databases with an “and” term between each of them. Other terms associated with ASD, such as Rett Syndrome, Pervasive Developmental Disorder Not Otherwise Specified, and Child Disintegrative Disorder were not included because they did not contribute to the overall number of studies found. The inclusion criteria were peer-review, English language, and published between 2000 and 2017. While research has been conducted before the year 2000, the purpose of this scoping review was to identify the most recent and relevant articles, and therefore the authors opted to exclude older publications.

Articles must have measured PA and recorded SF during participation in the research (see Appendix A for the list of inclusion and exclusion criteria). In accordance with the Compendium of Physical Activities (Ainsworth et al., 2000), the definition of PA was narrowed to include bicycling, conditioning exercises, dancing, running, sports, walking, and water activities. Studies were excluded if they did not fall under one of these categories. Based on the positive long-term mental and physical quality of life outcomes of leisure-time PA (Sanchez-Villegas et al., 2012), the researchers were interested in further exploration of recreational activities. Therefore, therapeutic interventions (i.e., physiotherapy, occupational therapy, therapeutic horseback riding) were excluded from the study. In addition, review articles and all other secondary sources were excluded from the study to ensure the analysis of primary data.

#### Databases

Four databases were utilized in this review based on topic area: SPORTdiscus (sports and recreation research), ERIC (research in education), PsycINFO (psychological research), and Medline (research in medical interventions). The researchers believed these four search databases would reach all the relevant journals within the area of interest. Overall, 1,168 articles were found using the above search terms and databases.

### Stage 3: Study Selection

**Figure 1:**
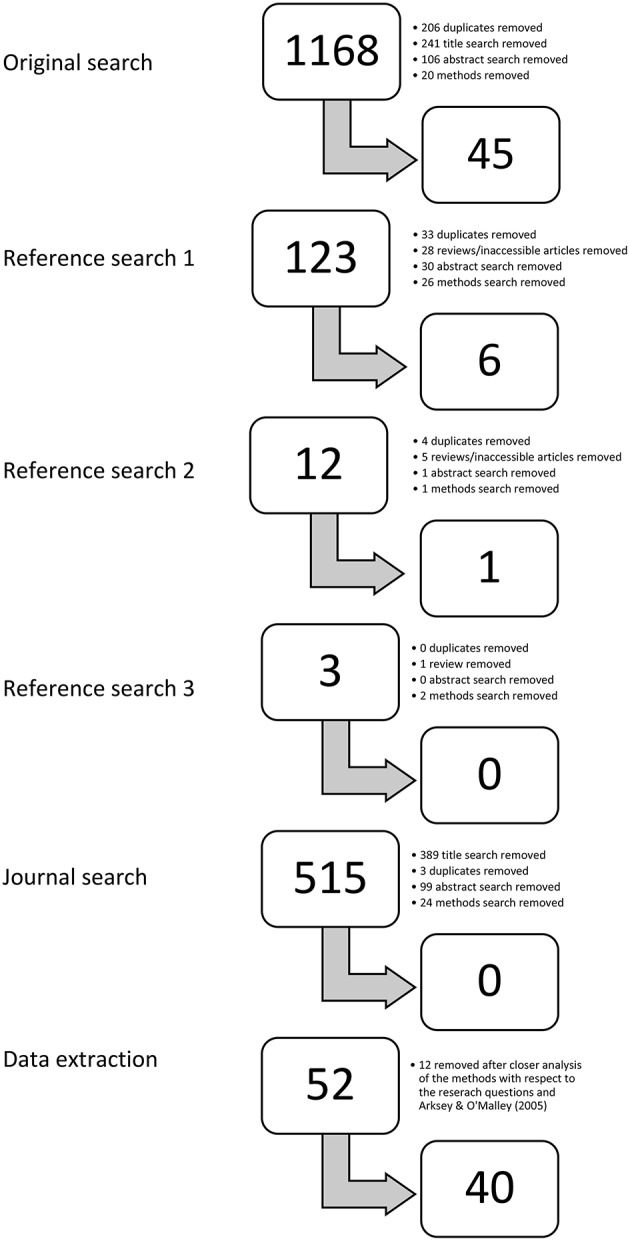
Study selection for the scoping review. Authors one and two reviewed a total of 1,699 (962+90+9+123+515) articles and found 52 suitable for the review of ASD, PA, and SF. Twelve articles were later removed because they did not fit within the inclusion criteria upon closer examination (*n* = 40). The methods were adapted from Arksey and O'Malley (2005).

Duplicated titles between the four databases were removed (*n* = 206) leaving 962 articles to consider. The second author read the titles of all 962 articles to remove clearly irrelevant articles (e.g., those pertaining to animal models or genetic testing), reducing the total number to 221. The first two authors read the remaining abstracts independently and removed those clearly irrelevant (*n* = 106), leaving 115 articles for full text review. Any abstracts the authors disagreed upon were included at this point in the review process. The next step was to read the entire article, focusing primarily on the methods section. Again, the first two authors evaluated the articles separately, which resulted in 45 articles to include in the review. On average, the authors had a 76% agreement rate after reading the full articles. Any articles the first two authors disagreed upon were reread by both individuals with the feedback of the other author in mind (e.g., why she chose to include or discard a particular article). If the authors still disagreed after rereading the article, author five was consulted for a final decision. This only happened for two articles, which were then removed from the review. The total number of articles included in this scoping review was 45 (see Figure 1).

To ensure no articles were missing, relevant titles from the reference lists of these 45 articles were examined in the same way described above (title, abstract, full text) and six more were found relevant to the study (see Figure 1). The references of these six articles were also reviewed, and three seemed pertinent to the scoping review. The same process was taken to review these three articles, which resulted in one additional article, and none of the references from this article were pertinent to the review. The authors also applied the search terms in the Research in Autism Spectrum Disorders journal, as it was the most common source of articles from the 45 included. The same process was completed again (title, abstract, full text), but no articles fit within this scoping review (see Figure 1). This selection procedure resulted in 52 primary research studies to be included in the scoping review (45+6+1).

### Stage 4: Chart the Data

The fourth stage of the scoping review framework was to organize the data from the selected articles. Microsoft Excel was utilized for this stage. The collected data points were author(s), title, publication year, country of first author's affiliated university, research setting, purpose, participant demographics, research methods, measures, interventions, key findings, and limitations. The authors, participants, measures, interventions, and findings are summarized in Table 1. Aggregate data have been presented in the results section. As the first author compiled the data for Table 1, 12 articles were removed from the study because they were not primary research (*n* = 3), measured stereotypy and not SF (*n* = 5), did not isolate PA from other activities (*n* = 1), did not include a PA component (*n* = 2), or did not include a SF component (*n* = 1). These discarded articles were approved by author two and five before the analysis was completed.

**Table 1:** Data extraction from *n* = 40 articles included in the scoping review.

	**Participants**			**PA**		**SF**		**Findings**	
**Author(s)/Year**	***n*, M_**age**_, range**	**% Males**	**ASD type**	**Measure**	**Intervention**	**Measure**	**Intervention**	**Specific findings**	**General summary**
Alexander et al., 2011	*n* = 1, age = 15 years	100%	ASD	None	Social Skills and Sports Program (90 min, 2x/week, 14 weeks), focus on soccer	Skill rating form (parent-report), interview with parents, observations of target social skill behaviors	Social Skills and Sports Program (90 min, 2x/week, 14 weeks focus on soccer	Social Skills and Sports program led to increased social skills	PA/SF training → ↑ SF
Ayvazoglu et al., 2015	*n* = 6, M_age_ = 7.5 years (range = 4–13)	66.7%	HFASD	Accelerometers worn for 7 consecutive days	None	Q-sort survey and follow-up interviews with parents	None	Individuals with ASD less active because of social barriers	↓PA ∝↓SF
Bingham et al., 2015	*n* = 8, M_age_ = 10.88 years (total range = 8–16)	100%	ASD	SOCARP^a^ (during recess over 14 school days), accelerometers (worn 7 consecutive days)	None	SOCARP^a^ (during recess over 14 school days)	None	Most PA time spent alone for individuals with ASD	↓SF ∝↓PA
Bock, 2007	*n* = 4, M_age_ = 9.6 years (range = 9–10)	100%	AS	Observed time spent playing organized games (e.g., kickball)	None	Observation of social behaviors during games	Stop, Observe, Deliberate, Act (SODA)	SODA caused increased socialization during recess PA	SF training → ↑SF and PA
Boddy et al., 2015	*n* = 17, total M_age_ = 9.97 years (total range = 5–15)	81.4% (total)	ASD	Accelerometers worn for 7 consecutive days	None	SOCARP^a^ during recess	None	Children with ASD were less active when in small groups than they were playing on their own	↓SF ∝↑PA
Bremer et al., 2015	*n* = 9, M_age_ = 4.32 years (range = 4)	88.9%	ASD	None	Fundamental movement skills training (group 1: 60 min, 1x/week, 12 weeks; group 2: 6 min, 2x/week, 6 weeks)	Social Skills Improvement System, VABS-2^b^ (both parent-report), observation of play	None	Fundamental movement skills training led to increased SF	PA training → ↑SF
Cavanaugh and Rademacher, 2014	*n* = 11, M_age_ = 12.9 years (range = 10–16)	81.8%	ASD	SURF^c^ Camp Curriculum Activity Observation Checklist	Learning through Sun, and SURF: included surfing, yoga, group games (within 2 days, details not provided)	Social Skills Improvement System (parent-report), SURF^c^ Skills Checklist, SURF^c^ Camp Curriculum Activity Observation Checklist	SURF^c^ Social Skills Curriculum taught in a classroom one week prior to camp (details not provided)	SURF^c^ curriculum taught social functioning	PA/SF training → ↑ SF
Chu and Pan, 2012	*n* = 21, M_age_ = 8.72 years (range = 7–12)	95.2%	ASD	None	Peer or sibling assisted swimming (60 min, 2x/week, 16 weeks)	Observation of physical and social behaviors	Peer or sibling assisted swimming (60 min, 2x/week, 16 weeks)	Swimming with peers/siblings had higher social interactions than controls	PA/SF training → ↑ SF
Ferguson et al., 2013	*n* = 9, M_age_ = 8.33 years (range = 7–11)	100%	ASD	None	Small group social skills and sportsmanship training (90 min, 1x/week, 10 weeks)	Observation of sportsmanship and social skills	Small group social skills and sportsmanship training (90 min, 1x/week, 10 weeks)	Wii Sports and social training increased social skills and sportsmanship	PA/SF training → ↑ SF
Hilton et al., 2008	*n* = 52, M_age_ = 9.54 years (range = 6–12)	84.6%	HFASD	Scale of PA participation intensity over the past 4 months (parent-report)	None	Scale of with whom PA was participated over the past 4 months (parent-report)	None	Less diversity with whom youth with ASD participate in PA	↓PA ∝↓SF
Karakaş et al., 2016	*n* = 36, M_age_ = 5.36 years (range = 4–6)	69.4%	ASD	Hours of exercise (reported by physical educator)	None	Social Skills Evaluation Scale, Ladd and Profilet Child Behavior Scale (both educator report)	None	More PA time related to more positive social skills in children with ASD	↑PA ∝↑ SF
Ketcheson et al., 2018	*n* = 20, M_age_ = 4.96 years (range = 4–6)	75%	ASD	Accelerometers worn for 7 consecutive days at 3 time points (1-week pre-intervention, 1-week post-intervention, 4-weeks post-intervention)	Motor skill intervention (4 h, 5x/week, 8 weeks)	Playground Observation of Peer Engagement, Mullen Scales of Early Learning, VABS-2^b^ (parent-report)	None	Less time spent in solitary play after the motor skill training, no differences in PA at any test point	PA training → ↑ SF
Ledford et al., 2016	*n* = 2, M_age_ = 4.63 years (range = 55–56 months)	100%	ASD	Accelerometers worn during first 10 min of recess, 4 days/week	1:1 low- and high-effort social and PA intervention over 36 recess sessions	Direct Assessment Tracking Application during first 10 min of recess, 4 days/week	1:1 low- and high-effort social and PA intervention over 36 recess sessions	Improved SF, but PA results varied between the two participants	PA/SF training → ↑SF, but ?PA
Loy and Dattilo, 2000	*n* = 1	100%	AS	Video and direct observation of game play	Competitive and cooperative games (10 min, 3x/day, 2days/week, 14 weeks)	Video and direct observation of social interactions	None	Cooperative games > competitive games >> free play regarding positive and negative social interactions	PA training → ↑SF
MacDonald et al., 2017	*n* = 9, M_age_ = 5.18 (range = 2–7)	88.9%	ASD	None	Motor-play with parent/caregiver (10 min)	Video observation using Early Head Start 24-Month 3-Bag Scales	Social-play with parent/caregiver (10 min)	More positive and less negative socialization in social-play than in motor-play (e.g. PA)	social-play SF > motor-play SF
Macpherson et al., 2015	*n* = 5, M_age_ = 10.7 years (range = 9–11)	80%	ASD	None	Kickball (13–21 sessions, details not provided)	Video observation of social behaviors	Video clip demonstrating positive reinforcement	Children demonstrated compliment gestures during kickball after watching a video	SF training → ↑SF in PA
Magnusson et al., 2012	*n* = 6, M_age_ not provided (range = 9–15)	66.7%	ASD	None	1:1 exercise training (60 min, 2x/week, 16 sessions)	Survey (parent–report)	None	Social skills increased after exercise program	PA training → ↑SF
Matsushita and Sonoyama, 2010	*n* = 1, age 11 years	100%	AS	Video observation of throwing skills	Target throwing practice (60 min, 2x/month, 17 sessions)	Video observation of participant skills and comments, conversation with mother	None8	Improved throwing skills led to improved confidence and interest for engaging in PA with others	PA training → ↑SF in PA
Memari et al., 2017	*n* = 68, M_age_ = 9.8 years (range = 6–16)	61.8%	ASD	Accelerometers worn for 7 consecutive days	None	Autism Social Skill Profile	None	Young people with ASD who had higher SF gained more PA	↑SF ∝↑PA
Memari et al., 2015	*n* = 83, M_age_ = 9.8 years (range6–15)	63.8%	HFASD	GSLTQ^d^ (modified) (parent-report)	None	GSLTQ^d^ (modified) (parent-report)	None	Decreased social play associated with decreased SF	↓PA ∝↓SF
Miltenberger and Charlop, 2014	*n* = 3, M_age_ = 8.17 years (range = 6–9)	66.7%	Autism	Video observation of participation	10 min of 1:1 athletic skill training then 10 min of 1:1 rules training, both for handball and 4-square	Video observation of social skills	None	Skill and rules training increased speech and appropriate group play participation	PA training → ↑SF and ↑PA
Movahedi et al., 2013	*n* = 30, M_age_ = 9.13 years (range = 5–16)	86.7%	ASD	None	Kata training (90 min, 2x/week, 14 weeks)	GARS-2 (parent report)	None	Kata training led to increased social interaction and decreased social dysfunction	PA training → ↑SF
Must et al., 2015	*n* = 53, M_age_ = 6.6 years (range = 3–11)	83.5%	ASD	Questionnaire	None	VABS-^b^ (parent report)	None	Social skills reported as a barrier to PA	↓SF ∝↓PA
Obrusnikova and Cavalier, 2011	*n* = 14, M_age_ = 10.64 years (range = 8–14)	85.7%	ASD	Accelerometers worn for 5 weekdays and 2 weekend days over a 14-day period	None	Social Responsiveness Scale (parent report), photos, questionnaire, 1:1 interview	None	Social skills were barriers to and facilitators of PA (e.g., having a friend for PA)	↓SF ∝↓↑PA
Obrusnikova and Miccinello, 2012	*n* = 103, M_age_ = 12 years (range = 5–21)	82.5%	ASD	Questionnaire (parent report), focus groups with parents	None	Questionnaire (parent report), focus groups with parents	None	Both advantages (e.g., practice social skills) and disadvantages (e.g., getting teased) of PA	↑PA ∝↓↑SF
Pan and Frey, 2005	*n* = 30, M_age_ = 13.2 years (range not provided)	90%	ASD	Accelerometers worn for 7 consecutive days, questionnaire (youth report), survey (parent report)	None	Questionnaire (youth report), survey (parent report)	None	Parental PA did not influence PA of youth with ASD	SF */*⊂ PA
Pan, 2009	*n* = 25, M_age_ = 9.28 years (range = 7–12)	100%	ASD	Accelerometers worn for 5 weekdays during school time	None	Engagement Check observation	None	PA and SF in Phys. Ed. > recess, peer socialization did not affect PA, adult support increased PA in Phys. Ed. only	SF */*⊂ PA
Pan, 2010	*n* = 16, M_age_ = 7.23 years (range = 6–9)	100%	HFASD	None	Water exercise swimming program (90 min, 2x/week, 10 weeks)	School Social Behavior Scales (teacher report)	None	Various social behaviors improved after participating in the swim program	PA training → ↑SF
Pan et al., 2011	*n* = 19, M_age_ = 14.19 years (range not provided)	100%	ASD	Accelerometers worn during two physical education classes	None	Video observation of physical education	None	Social initiations and interactions related to PA with peers, but not with adults	↑SF ∝↑PA
Potvin et al., 2013	*n* = 30, M_age_ = 9.25 years (range = 7–13)	86.7%	HFA	Children Assessment of Participation and Enjoyment/Preference for Activities of Children	None	GARS-2^e^, VABS-2^b^ (both parent report) the Test of Nonverbal Intelligence, 3rd ed., Comprehensive Assessment of Spoken Language	None	No difference in social aspect of PA between TD and ASD	PA */*⊂ SF
Radhakrishna, 2010	*n* = 6 (range = 8–14)	83.3%	ASD	None	Yoga (45 min, 5x/week, 10 months)	Questionnaire (parent report)	None	Improved social-communication skills after yoga intervention	PA training → ↑SF
Schenkelberg et al., 2015	*n* = 6, total M_age_ = 5.4 years (range = 5–6)	100%	ASD	Observational System for Recording Activity of Children-Preschool Version during summer camp	None	Behavioral Risk Factor Surveillance System, National Survey of Children's Health with Special Health Care needs (parent report)	None	Children with ASD more likely to be solitary during PA than with a peer or adult	↑SF ∝↓PA
Schenkelberg et al., 2017	*n* = 6, total M_age_ = 5.4 years (range = 5–6)	100%	ASD	Observational System for Recording Activity of Children—Preschool Version during summer camp	None	Behavioral Risk Factor Surveillance System, National Survey of Children's Health with Special Health Care needs (parent report)	None	Children with ASD more active during solitary play than with a peer, group, or adult	↑SF ∝↓PA
Smith, 2015	*n* = 13, M_age_ = 10.3 years (range = 8–11)	0%	ASD	Pedometers worn for 7 consecutive days at 3 time points	Multi-Sport Camp (8 h/day, 5 days)	VABS-2^b^	None	Increased social skills after the multi-sport camp	PA training → ↑SF
Solish et al., 2010	*n* = 65, M_age_ = 9.9 years (total range = 5–17)	87.7%	ASD	Questionnaire (parent report)	None	Questionnaire (parent report)	None	Recreational PA with peers: ASD < ID < TD	↓SF in PA
Sutherland and Stroot, 2010	*n* = 1, age = 13 years	100%	ASD	None	3-day rock climbing trip (8 h of rock climbing)	Observations, checklists (participant report), interviews with participants	Team building activities	Team building contributed to integration for youth with ASD, but he made little effort to socialize in PA	SF training → ↑SF during PA, PA */*⊂ SF
Tan, 2011	*n* = 12, M_age_ = 4.86 years (range = 2–6)	100%	ASD	None	Tri-cycling (15 min, 8 sessions)	Pediatric Quality of Life Inventory (parent report)	None	Tri-cycling group had higher social scores than control group	PA training → ↑SF
Tint et al., 2017	*n* = 66, M_age_ = 16.63 (range = 11–22)	75.8%	ASD and ID	Participation and Environment Measure for Children and Youth (parent report)	None	Participation and Environment Measure for Children and Youth (parent report)	None	Social demand of PA is a barrier for participation, relations with peers is a barrier and facilitator	↑SF ∝↓↑PA
Ward and Ayvazo, 2006	*n* = 2, age = 8 years	100%	Autism	Video observation of physical education	Classwide Peer Tutoring in physical education (30 min, 2x/week, 13 weeks)	None	Classwide Peer Tutoring physical education (30 min, 2x/week, 13 weeks)	Improved PA engagement after classwide peer tutoring	PA/SF training → ↑PA
Zachor et al., 2016	*n* = 51, M_age_ = 5.33 years (range = 3–7)	78.4%	ASD	None	Outdoor challenge-based activities (30 min, 1x/week, 13 weeks)	Social Responsiveness Scale, VABS-2^b^, Teacher's Perceived Capabilities Questionnaire (all teacher report)	None	Improved social skills after Outdoor Adventure Program	PA training → ↑SF

*Key findings about PA and SF was summarized. ↑ (increased) → (affected by) ∝ (non-causal relationship) /⊂ (unclear relationship)*.

a *SOCARP (System for Observing Children's Activity and Relationships during Play)*,

b *VABS-2 (Vineland Adaptive Behavior Scales−2nd edition)*,

c *SURF (Stay in the group; Use my SEE steps, Remember to ask questions, Form a friendship)*,

d *GSLTQ (Godin-Shephard Leisure Time Questionnaire)*,

e *GARS-2 (Gilliam Autism Rating Scale−2nd edition). Study type: purple (PA intervention), blue (PA/SF intervention), red (SF intervention), green (cross-sectional)*.

### Stage 5: Collate, Summarize, and Report Results

The last stage of the Arksey and O'Malley (2005) framework was to organize the relevant findings into themes, prioritizing the results based on relevance to the research questions and focusing heavily on the intervention type. Pertinent data, such as sample size, participants, methods, and outcomes were included. All data have been reported in the results section below.

#### Ongoing Consultation

Arksey and O'Malley (2005) suggested a scoping review should include the consultation of experts in the area of research. For this review, three professors (including authors four and five) and five graduate students in the Department of Kinesiology at Wilfrid Laurier University were consulted during the development of the research questions, search terms, and inclusion criteria. The first and third author were enrolled in a university course centered on scoping review methodology, where they consulted with one professor and five graduate students during class time each week for three months. Authors four and five were the project advisors, and they were consulted as needed throughout the review process. The advisors approved the research questions, search terms, and inclusion criteria before the review was conducted. They also reviewed the data extraction excel file and met with author one to discuss their interpretation of the results.

## Results

### Study Demographics

**Figure 2:**
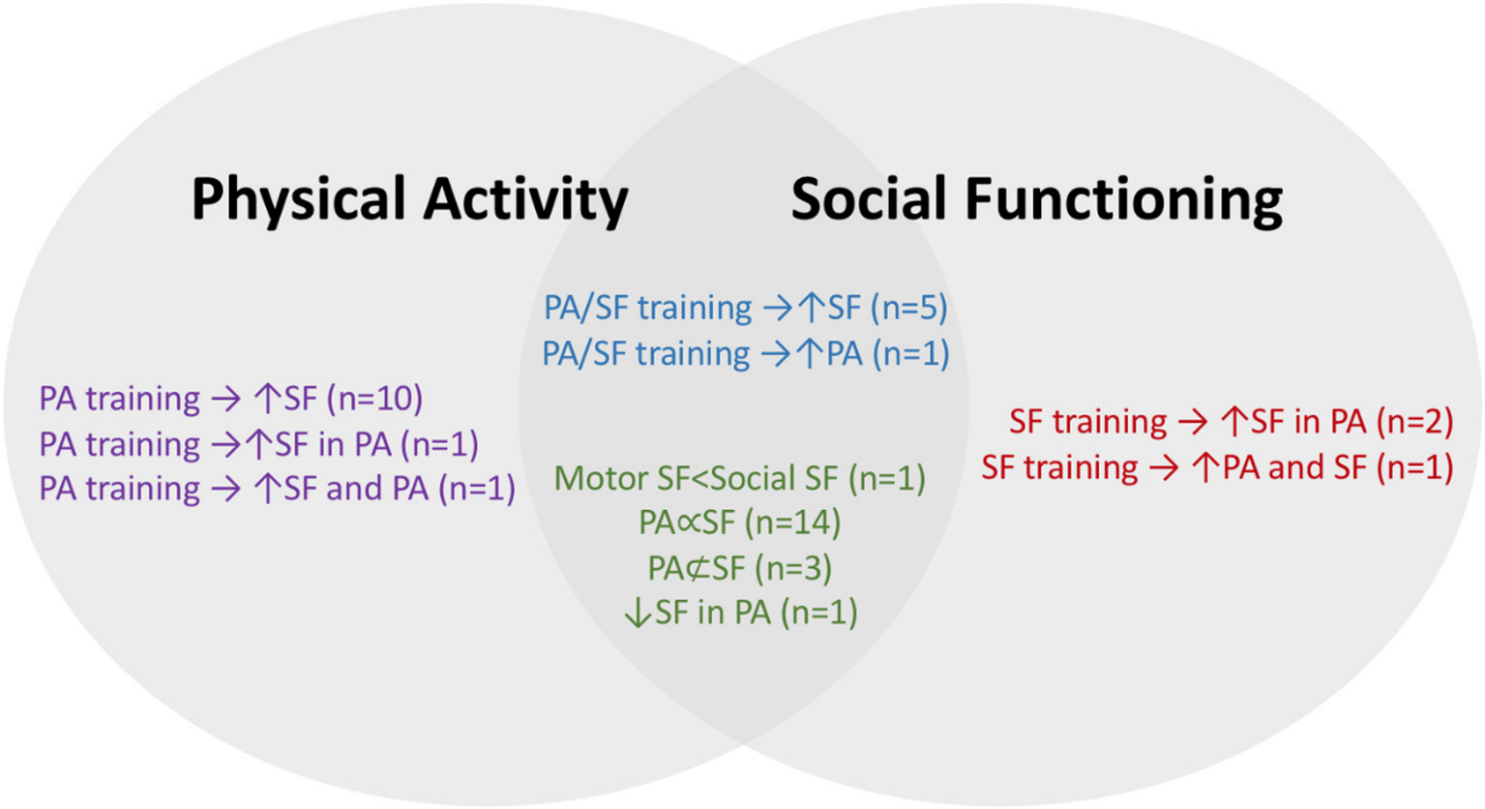
Visual representation of general summary. The key findings about PA and SF were summarized (*n* = 40). Study type: purple (PA intervention), blue (PA/SF intervention), red (SF intervention), green (cross-sectional). Symbol key: ↑ (increased), → (affected), ∞ (non-casual relationship), */*⊂ (unclear relationship).

Following the Arksey and O'Malley (2005) framework and the inclusion criteria outlined above, 40 peer-reviewed articles were found relating to the research purpose (see Table 1 and Figure 2). Half of the articles (*n* = 20) were published between 2014 and 2017 and published in the United States. Considering demographics, 30 articles identified participants as having an ASD diagnosis (75.0%), five high functioning ASD (12.5%), three Asperger's syndrome (7.5%), and one Autism (2.5%). Of the participants with ASD, the mean age was 9.1 years (ranging from 4.32 to 16.63 years) and the average sample size was 23 participants (ranging from 1 to 103). Males made up 86.1% of the participants, ranging from 0 to 100%.

The majority of studies were conducted in a community setting (*n* = 29, 72.5%), while ten (25%) took place at a school and one (2.5%) both in the community and at a school. Twenty-four articles employed interventions to increase PA (e.g., jogging, swimming, etc.) and ten to increase SF (e.g., social skills training, teambuilding exercises, etc.). Nine studies included both PA and SF interventions (e.g., a social skills and sports program), while 15 did not include an intervention, but examined PA and SF at one point in time (e.g., parent report questionnaire assessing PA and SF). Table 1 outlines details about interventions and data collection methods.

### Cross-Sectional Studies

Based on the inclusion criteria (Appendix A), each study explored PA and SF together in some way. The most common finding was “PA ∞ SF” (*n* = 14, 35.0%, coded in green on Table 1), meaning there was a relationship between PA and SF determined from a cross-sectional means of data collection, such as a questionnaire or survey. Of these 14 articles, eight demonstrated positive relationships where PA and SF increased or decreased together. For instance, Memari et al. (2015) found that social-play decreased with lower SF in children with HFASD (M_age_ = 9.80 years) (↓PA∞↓SF). In a subsequent paper, Memari et al. (2017) reported higher activity levels in children with ASD (M_age_ = 9.80 years) if they had higher social functioning scores (↑PA∞↑SF). Conversely, three studies showed that PA increased as SF decreased, or vice versa. Boddy et al. (2015) found that children with ASD (M_age_ = 9.97 years) were less active when with peers than in solitary play (↑PA∞↓SF). Similarly, the studies by Schenkelberg et al. (2015) and Schenkelberg et al. (2017) demonstrated more activity during solidary play than PA with a peer or adult (↓PA∞↑SF) in children (Mage = 5.4 years).

Three studies showed a mixed relationship between PA and SF, such as Obrusnikova and Cavalier (2011), who found that PA was associated with social advantages (e.g., opportunity to gain social skills) and disadvantages (e.g., bullying) in youth (M_age_ = 10.64 years) with ASD (↑PA∞*↓↑*SF). Similarly, it was highlighted by Tint et al. (2017) that social demands were both a barrier to and facilitator of PA for youth (M_age_ = 16.63 years) with ASD and intellectual disability (↑SF ∞*↑↓*PA). Taken together, these 14 studies demonstrated the importance of considering both PA and SF for people with ASD, as some social factors were positively associated with PA and others were negatively associated.

Contrary to the studies above, three articles found “PA */*⊂ SF”, meaning there was not a clear relationship between PA and SF determined from a cross-sectional means of data collection. Pan and Frey (2005) reported no influence of parental PA (which was the SF in this case) on the PA of their youth with ASD (M_age_ = 13.2 years). Pan's study (2009) demonstrated that the PA of children with ASD (M_age_ = 9.28 years) during recess did not change based on social engagement with peers, but children were more active when with adults. Finally, Potvin et al. (2013) found no differences in social engagement during PA between children with high functioning Autism (M_age_ = 9.25 years) and their typically developing peers. These three studies suggest a complexity in the way SF is related to PA for people with ASD.

One article discussed the status of social interactions within recreational PA (↓SF in PA). Solish et al. (2010) discovered that young people with ASD (M_age_ = 9.9 years) were involved in significantly fewer recreational activities with peers than young people with intellectual disabilities or TD; however, there was no difference in recreational activity participation with parents or adults between the three groups. Young people with ASD participated in significantly fewer social and recreational activities than their TD peers overall. Further, participants with ASD had fewer mutual friends than the other two groups; however, the cross-sectional nature of the study does not indicate whether that was the reason for or outcome of reduced recreational PA with peers (Solish et al., 2010).

MacDonald et al. (2017) found that children with ASD (M_age_ = 5.18 years) engaged in more positive and less negative socializing in social play settings (e.g., strings, cars, blocks, etc.) with their parents as compared to motor-skill play settings (e.g., mini-stairs, balance beam, tricycle, etc.). The socialization behaviors of children with ASD were significantly worse than TD children in three out of four measures during motor play, but only one out of four measures during social play. The findings from this study were coded as “social-play SF > motor-play SF” in Table 1. Taken together, the research by Solish et al. (2010) and MacDonald et al. (2017) shed light on the social behaviors of young people with ASD in different activities.

### PA Intervention Studies

The second most common finding (*n* = 10 studies) was “PA training → ↑SF”, meaning some form of PA intervention was associated with increased SF at post-testing. PA interventions included fundamental motor skills training (12 h), motor skill intervention (160 h), competitive and cooperative games (14 h), 1:1 exercise training (32 h), kata (e.g., martial arts) training (42 h), water exercise swimming program (30 h), yoga (~150 h), Multi-Sport camp (40 h), tri-cycling (2 h), and outdoor intervention program (6.5 h). Each of these interventions caused an increase in social functioning for the young people with ASD who participated. These studies are coded in purple on Table 1.

One study found that PA not only increased SF, but also influenced PA (PA training → ↑SF and PA). Miltenberger and Charlop (2014) practiced the skills necessary for four-square and handball for 10 min with three children with Autism (M_age_ = 8.17 years), and then another 10 min teaching the rules for each game. Participants demonstrated increased functional speech (SF) and appropriate group play participation (PA) in both games after the training procedures. The participants retained these skills for up to 16 weeks after the intervention; however, these gains did not translate to other recess activities.

The findings of Matsushita and Sonoyama (2010) are akin to those of Miltenberger and Charlop (2014), who observed an SF outcome specific to their PA intervention (PA training → ↑SF in PA). An 11-year-old male with Asperger syndrome participated in 17 one-hour sessions of target throwing practice with the researcher. As his ball throwing skills improved, the participant demonstrated more confidence in his ability to participate in physical activities and expressed interest in playing sports with others. His mother reported that he played catch with his father and brother at home more as a result from the target throwing intervention. In this way, the PA intervention improved his interest in engaging with other people to demonstrate his newfound skill (Miltenberger and Charlop, 2014).

### PA/SF Intervention Studies

Six studies employed an intervention that included both a PA and SF component (coded in blue on Table 1), four of which resulted in increased SF (PA/SF training → ↑SF). Alexander et al. (2011) led to increased social skills for a 15-year-old male with ASD in the program, in other leisure programs, at school, and in day-to-day life. Similarly, youth with ASD (M_age_ = 7.33 years) who participated in a social skills and sportsmanship training program displayed more positive behaviors (e.g., giving compliments) and fewer negative behaviors (e.g., making negative comments) after ten 90-min sessions (Ferguson et al., 2013). Youth with ASD (M_age_ = 12.9 years) who participated in the Learning through Sun, Sand, and SURF program demonstrated increased interaction, confidence assertion, responsibility, and engagement at post-testing (Cavanaugh and Rademacher, 2014). Last, young people with ASD (M_age_ = 8.72 years) who participated in a swimming program with peer-assistance or sibling-assistance made more social gains than those who participated in the regular adult-assisted swimming program. These four studies demonstrate that PA may be a medium for the development of social skills for young people with ASD.

The article by Ledford et al. (2016) combined PA and SF training and found improvements in SF similar to the four studies above, but opposing outcomes regarding PA (PA/SF training → ↑SF but ?PA). Two males with ASD (M_age_ = 4.63 years) received one-on-one support from an adult during recess at three different levels of interaction: (1) low-effort interaction, such as reminding participants about the available toys; (2) high-effort interaction, like offering choices between two toys and assisting with toy set up for play; or (3) enhanced intervention, in which the child received verbal and physical prompts to engage in PA. The high-effort and enhanced interventions were most effective for improving SF and PA as compared to low-effort and baseline. Both participants displayed improved engagement, proximal play, and peer interaction; however, only one of the two participants was more active. (Ledford et al., 2016).

Conversely, the study by Ward and Ayvazo (2006) utilized social interactions in physical education to encourage two eight-year-old boys with ASD to become more engaged in the class, which was coded as “PA/SF training → ↑PA”. Specifically, the boys with ASD were partnered with a TD peer in physical education who was taught to encourage participation and praise appropriate behavior. Despite variable results, both participants were more engaged in throwing and catching practice when working with their peers than they were during whole group instruction (Ward and Ayvazo, 2006). Between these six articles, it appears interventions utilizing PA and SF components had positive outcomes, particularly regarding SF, but potentially regarding PA as well.

### SF Intervention Studies

Three of the 40 articles in this review employed a SF intervention that contributed to SF and/or PA. These studies are coded in red on Table 1. Bock (2007) observed the socialization patterns of four children with Asperger's syndrome (M_age_ = 9.6 years) during class, lunch, and recess play after they participated in a Stop, Observe, Deliberate, Act (SODA) intervention. All four boys spent markedly more time playing organized sports at recess after eight SODA sessions and these outcomes were maintained during a five-month washout period (SF training → ↑PA and SF). The participants were also more cooperative during classroom activities and engaged with peers during lunch (Bock, 2007).

Macpherson et al. (2015) instructed youth with ASD (M_age_ = 10.7 years) to watch a video demonstrating how to give compliments to peers while playing kickball and found that the five participants employed these skills as a result, but only within the kickball setting (SF training → ↑SF in PA). More specifically, the participants gave verbal compliments, but they did not employ the gestures demonstrated in the videos (Macpherson et al., 2015). In the third SF intervention, Sutherland and Stroot (2010) observed a 13-year-old male with ASD as he engaged with his peers during a three-day rock climbing trip. Interestingly, it was not rock climbing that contributed to his belonging in the group (PA*/*⊂SF), but rather team building activities (SF training → ↑SF in PA) (Sutherland and Stroot, 2010).

### Study Limitations

Of the 40 studies included in this review, 34 listed at least one limitation in the discussion section of the article (see Appendix B), while most studies (*n* = 29, 72.5%) listed two, three, or four limitations. The most common limitation was a small sample size (*n* = 18), followed by missing measures (*n* = 13), meaning the authors felt additional measures would have enhanced their research, and sample generalizability (*n* = 12), meaning sample heterogeneity was low (e.g., a sample of all males). The six articles that did not list limitations were (1) Karakaş et al. (2016), (2) Magnusson et al. (2012), (3) Miltenberger and Charlop (2014), (4) Movahedi et al. (2013), (5) Pan et al. (2011), and (6) Radhakrishna (2010). For more information regarding these limitations, see Appendix B. Of note, the limitations listed in Appendix B are only those addressed in each individual article, and therefore there is a risk for bias if the authors of any articles included in the review did not include all the true limitations to their studies.

## Discussion

This scoping review examined 40 peer-reviewed research articles that incorporated PA and SF for young people with ASD. The primary finding from this analysis was the complex relationship between PA and SF for young people with ASD. The relationship appears to be bidirectional in the majority of studies; however, contextual factors were found to be highly influential as well. These findings are supported by the model by Van der Ploeg et al. (2004), regarding the role of personal and environmental factors in PA for people with disabilities, specifically the social stimulation during PA for young people with ASD (Obrusnikova and Miccinello, 2012). Several aspects of this review should be examined in depth as they pertain to characteristics of ASD (e.g., social and behavioral deficits) as well as the current literature.

From the cross-sectional articles, there was conflicting evidence regarding the interaction of people with ASD and their peers in PA. Some studies reported positive peer influence on PA participation (Pan et al., 2011), while others did not (Pan, 2009; Sutherland and Stroot, 2010). Further, young people with ASD were more active when alone than they were with small groups of peers (Boddy et al., 2015), and most of this PA was locomotor in nature (e.g., walking) (Bingham et al., 2015). Perhaps young people with ASD were more active on their own due to restrictive and repetitive behaviors (Leekam et al., 2011) rather than structured PA. Potvin et al. (2013) reported no differences between the social interactions of TD children and children with ASD during PA; however, Hilton et al. (2008) found lower diversity of social interactions during PA for young people with HFASD than those with TD. For example, Solish et al. (2010) reported that young people with ASD not only engaged in fewer recreational activities with peers than TD young people, but also less than young people with intellectual disabilities.

One suggested reason for the conflicting findings in PA is due to the elevated social stimulation experienced in group settings such as physical education. For instance, Healy et al. (2013) interviewed 12 children aged nine to 13 with ASD about their experiences in physical education. Of the emergent themes, individual challenge including physical inability, sensory issues, and fear of injury, influenced the children's experiences in physical education. Moreover, peer interactions were both positive (e.g., teamwork, friendship) and negative (e.g., bullying, social comparisons) in a particular setting. The children were excluded from PA at times due to personal choice (e.g., preference to watch), as well as peer choice (e.g., not welcomed to play because of inadequate skill) (Healy et al., 2013). Further, Eversole et al. (2016) examined activity enjoyment for children six to 13 years-old with ASD (*n* = 67) and found enjoyment in formal and informal activities was significantly higher in TD children. More specifically, age and ASD symptom severity were negatively correlated to physical and social activity enjoyment: as children grew older and/or had more severe symptoms, they were less likely to enjoy the activities. Clearly there are many social factors in group PA settings affecting the participation of children with ASD.

Many social facilitators and barriers of PA were highlighted in this scoping review, such as enjoyment, interest, and program availability; and bullying, lack of social skills, parent worry, respectively (Obrusnikova and Cavalier, 2011; Obrusnikova and Miccinello, 2012; Must et al., 2015; see Ayvazoglu et al., 2015). These positive and negative factors were shown to affect PA in young people with ASD, making the finding by Memari et al. (2017) understandable: those with higher SF were more active. One hypothesis is reduced socialization skills increase the barriers experienced by young people with ASD and therefore interfere with their interest in PA. There was conflicting evidence regarding the number of barriers and facilitators. For instance, all qualitative themes found in Ayvazoglu et al. (2015) were barriers to PA, both for children and parents. Must et al. (2015) also focussed entirely on barriers to PA rather than facilitators. Conversely, both studies by Obrusnikova and Cavalier (2011); Obrusnikova and Miccinello (2012) found a higher proportion of PA facilitators than barriers. These findings varied according to level, where intrapersonal barriers tended to outweigh facilitators, but barriers at interpersonal, physical, community, and institutional levels were similar or lesser than the facilitators. It is necessary for professionals, educators, and community members to minimize the barriers to and maximize the facilitators of PA for young people with ASD, not only to influence physical health, but also SF. For instance, leisure time PA has been associated with various positive social outcomes for TD adults such as social functioning and mental health (Sanchez-Villegas et al., 2012).

The intervention studies shed light on the various types and intensities of PA, which may address some of the barriers and facilitators. Several studies educated young people with ASD how to complete a particular PA skill (e.g., Miltenberger and Charlop, 2014), while others educated participants about how to socialize in the PA setting (e.g., Macpherson et al., 2015). Still others facilitated socialization to enhance PA (e.g., Alexander et al., 2011), as perhaps young people with ASD simply do not have social supports in PA and do not feel comfortable to participate (Healy et al., 2013). The intervention studies together suggest PA as a means for developing social skills, and perhaps people with ASD could develop these skills in a PA environment (Ferguson et al., 2013). On the other hand, training a child how to interact in a PA setting could improve the PA levels and positively affect health in a variety of other ways, not just social (Bock, 2007).

One intervention found a positive gain in PA and SF for young people with ASD who had periodic assistance from an adult during recess (Ledford et al., 2016). The interactions between young people and adults was more consistently influential than the interactions between peers. Pan (2009) reported that adult support (e.g., from an educator) positively influenced PA during physical education at school. Conversely, Pan and Frey (2005) found no effect of parental PA on their children with ASD, which has also been shown in TD children and their parents (Jago et al., 2010). Children with ASD have also reported more difficulties engaging in motor-play with their parents than with social or quiet play (MacDonald et al., 2017). The authors hypothesize that children with ASD are unsure of how to participate in PA settings or that the PA settings are too overwhelming. Further, maybe parents of children with ASD have similar challenges when engaging their children into PA as parents of TD children (e.g., limited time, resources, support, etc., see Martin Ginis et al., 2016). Perhaps the *role* of the adult is important, where physical educators seem to have a positive role (Pan, 2009), but parents seem to have a neutral role (Pan and Frey, 2005).

One-on-one PA interventions between individuals with ASD and adults present favorable conditions for social and motor improvements (Sowa and Meulenbroek, 2012). Toscano et al. (2018) organized participants (*n* = 46, M_age_ = 8.2) into exercise groups of no more than three child/caregiver dyads. Positive outcomes were reported in several domains, supporting the contention for small group size and high proportion of adults as most beneficial for young people with ASD when engaging in PA. However, these studies support segregation between young people with disabilities and their TD peers, which is contrary to arguments made by the social model of disability (Walsh-Allen, 2010). Perhaps classwide peer tutoring is one method to improve engagement in physical education while maintaining an integrated class environment (Ward and Ayvazo, 2006). Further, peer interactions in PA may demonstrate to young people with ASD how to socially engage in particularly settings (Pan et al., 2011).

### Future Directions

The findings from this review highlight an area of research that may contribute to the quality of life for people with ASD, and their families by extension. While none of the reviewed studies examined outcomes beyond several weeks, they provide evidence to support the undertaking of such research. Children with ASD demonstrated increased physical and psychosocial quality of life, as well as decreased autistic symptoms after a 48-week exercise program (40 min, twice per week) (Toscano et al., 2018). Perhaps the long-term effects of PA for people with ASD are similar to the effects for TD adults (Sanchez-Villegas et al., 2012). However, monetary and personnel resources are limited for many people with ASD (Must et al., 2015), which makes the undertaking of intensive long-term programming inaccessible. Fortunately, positive outcomes were found from interventions as short as 20 min (Miltenberger and Charlop, 2014), which has implications for the amount of resources allocated to programming for people with ASD. Conclusions about the dosage of intervention cannot be made due to the nature of this scoping review. Further, the methods of these studies do not lend themselves to a meta-analysis; thus, more rigorous outcome measures are needed to determine the ideal frequency and intensity of PA/SF interventions. Various intervention types had positive effects for young people with ASD, and therefore, PA/SF programming should be employed based on feasibility rather than length or intensity until more clear recommendations can be made from the literature.

Studies have demonstrated the diverse effects of PA on quality of life, not just SF (Srinivasan et al., 2014; Toscano et al., 2018). Perhaps the most effective way to apply all of this information is to educate young people with ASD about PA (e.g., how to perform a skill, how to socialize with peers) in small groups with one-on-one adult support. In addition, future research is needed to determine how to mitigate the negative social and motor outcomes of large group PA environments for people with ASD (e.g., Sowa and Meulenbroek, 2012), to avoid falling back into segregated programming. If the necessary supports are provided, perhaps PA could serve as an outlet for people with ASD in a variety of settings (e.g., school). Research has shown that PA reduces stereotypy (Lang et al., 2010) and may be a means of coping with social stressors (Müller et al., 2008) for people with ASD. The authors hypothesize, if PA is incorporated into early ASD interventions, it will positively influence the primary areas of concern (e.g., social skills and repetitive behaviors) associated with the disorder. Early intervention is key for creating life-long habits (Corsello, 2005; Leekam et al., 2011), and PA/SF training may be one method to holistically address the concerns of ASD, while also influencing life long-term quality of life.

Finally, those interested in conducting future research in the area of PA, SF, and ASD should consider the limitations highlighted in the 40 reviewed articles (see Appendix B). There appears to be a noticeable gap in the literature as no studies in this review included participants under the age of four and over the age of 16. Adults with ASD reported utilizing PA to cope with social stressors (Müller et al., 2008), and therefore may have different interactions with PA than young people. There was also a higher representation of participants with HFASD and Asperger's syndrome than participants with more severe cases of ASD. Social and motor skills decrease with increasing severity of ASD symptoms (Memari et al., 2015, 2017), meaning these findings may not apply to people with ASD who require very substantial support. Researchers should seek to understand whether there are differences across ASD levels of severity.

Males (86%) made up the majority of participants, which is unsurprising, as males are four times more likely to be diagnosed with ASD than females (Center for Disease Control and Prevention, 2018). Boddy et al. (2015) found no difference in PA between girls and boys, but Memari et al. (2015) found higher risk for inactivity amongst girls with ASD than boys (Memari et al., 2015). Other research shows increased influence of parents on the sedentary behaviors of TD girls than boys (Jago et al., 2010). Taken together, future research should examine how social influences affect the PA behaviors of females with ASD.

Much of the research was conducted in North America (*n* = 24) and Taiwan (*n* = 5). Due to varying cultural norms, the social impairments associated with ASD may be perceived differently from culture to culture. For example, while eye contact is recognized as socially appropriate in the North America, this may not be true around the world (Matson et al., 2012). Consequently, the lack of ethnic differences observed by Lindquist et al. (1999) regarding PA patterns suggests the importance of investigating other factors, for example family background and ethnic characteristics. Despite the potential differences in social deficits cross-culturally, it has been found that children and adolescents diagnosed with ASD have severe impairments in adaptive and appropriate social skills (Matson et al., 2012). In order obtain more comprehensive results, future research should explore factors influencing PA in a variety of locations and with participants who have diverse ethnic backgrounds.

### Limitations

The primary limitation of the scoping review methodology is the lack of quality assessment of the included articles. However, the goal of a scoping review is simply to identify research that has been conducted, not necessarily to assess quality. Arksey and O'Malley (2005) state “…the scoping study does not seek to assess quality of evidence and consequently cannot determine whether particular studies provide robust or generalizable findings.” (p. 27). While quality assessment was not a goal of the research, quality should be considered before applying these findings to interventions or programming for young people diagnosed with ASD.

Some limitations should be noted regarding the quality of this scoping review. While the first two authors took many steps to ensure all relevant articles were included in the review, it is possible some studies were missed due to the selection of databases and search terms. Second, the first two authors each conducted title reviews independently, meaning if one author determined an article was irrelevant based on the title, the other author would not have seen it. This was not the case however, during the abstract review, in which both authors assessed the abstracts to determine whether the full article would be read. To thwart the possibility for lost information, both authors erred toward inclusion during the title search and became more exclusive at later stages of the review, which is why 12 articles were later removed during the data extraction phase of the research. In terms of methodology, this review was limited to four databases and articles published in English since the year 2000. These criteria may have biased the results.

## Conclusion

In summary, this scoping review provides insight into the relationship between PA and SF in young people with ASD. From the current literature, PA may be related to the social interactions and behaviors of young people with ASD. This review has summarized the relevant literature regarding PA and SF and suggests future directions for research. It has become evident that PA is a viable intervention option to target some of the primary concerns associated with ASD. Further, interventions educating young people with ASD about how to engage in PA may enhance quality of life through increased PA participation and diversified social relationships.

## Author Contributions

NR created research questions, conducted article search, analyzed data, and wrote manuscript. AB conducted article search and contributed to manuscript editing. KW created research questions, contributed to manuscript writing, and editing. PF supervisor of research. PB supervisor of research.

### Conflict of Interest Statement

The authors declare that the research was conducted in the absence of any commercial or financial relationships that could be construed as a potential conflict of interest.
