# Supplementary material for: Scoping Review: Physical Activity and Social Functioning in Young People With Autism Spectrum Disorder
Source: Front Psychol. 2019 Feb 13;10:120. doi: 10.3389/fpsyg.2019.00120 (PMC6381857; doi:10.3389/fpsyg.2019.00120)
Supplement: Supplementary file 1 [file Data_Sheet_1.docx]

**Appendix A: Coding Manual**

**Inclusion Criteria**

- Physical activity: some description of PA in the methods section. This could have been an intervention (e.g., a swimming program, learn to bike program, etc.) or recreational (e.g., playground play, summer camp play, etc.). There needed to be a clear description of the PA, such as the frequency/intensity/type/time of PA, or step count recorded from an accelerometer.
  - Exclusions to this were therapeutic programs such as horseback riding or physical therapy, as the purpose of these activities are specific and are not generalizable to day-to-day PA.
- Social functioning: some description of SF in the methods section. This could have been social skills (e.g., ability to take turns, pro-social behaviours, etc.), planned social interactions (e.g., peer-tutoring interventions), or spontaneous social interactions (e.g., observed social interactions while on a playground). There needed to be a clear description of SF, such as a measurement like the Social Responsiveness Scale, or recorded time spent socializing with others.
  - Exclusions were socializing with one instructor (e.g., a one-on-one program between a child and instructor) because this was not enough of a social interaction as determined by the research group. Stereotypic behaviour was also excluded, because it was not a direct measure of social skills (however, it is related to socialization).
- Autism Spectrum Disorder: some description of ASD in the methods section. This could include Asperger’s Syndrome, Pervasive Developmental Disorder – Not Otherwise Specified, high-functioning Autism, etc. Papers were included if parents reported the ASD diagnosis, regardless of whether or not the authors discussed the DSM diagnosis procedure. In this way, a child could have been included whose parent indicated he/she had ASD but was not actually diagnosed by the DSM or a clinician.
  - Exclusions were studies that did not isolate participants with ASD from other participants, such as TD peers or those with a different disorder (e.g., Down Syndrome).

**Appendix B: Study Limitations**

Any limitations outlined within the discussion of the research article were extracted and summarized. Six studies did not list any limitations, but 34 listed up to seven limitations. The types of limitations were summarized into the 14 categories below.

**Definition, Frequency, and Example**

- Ceiling effects (n=1): Measures were not sensitive enough to capture changes of participants (e.g. Macpherson, Charlop, & Miltenberger, (2015)
- Analysis procedures (n=1): Statistical analysis based on the entire group rather than the two participants with ASD in question (e.g. Ward & Ayvazo, 2006)
- Lack of randomization (n=1): Participants were not randomly allocated to the control or intervention group (e.g. Zachor, Vardi, Baron-Eitan, Broadi-Meir, Ginossaur, & Ben-Itzchak, 2016)
- Participant attrition (n=3): Participants missed sessions of the intervention or did not engage in follow up testing (e.g. Ferguson, Gillis, & Sevlever, 2013)
- Limited application (n=3): Lack of background information and/or depth, therefore difficult to determine where else the findings may be applicable (Tint, Maughan, & Weiss, 2017)
- No control group (n=3): There was no control group of which to compare participants with ASD (e.g. Memari, Panahi, Ranjbar, Moshayedi, Shafiei, Kordi, & Ziaee, 2015)
- Convenience sample (n=8): The sample was recruited from one particular organization or location easily accessible by the researcher(s) (e.g. Pan, 2009)
- Reporting bias (n=8): Issues with data collection due to the participants’ recall of information or truthfulness (e.g. Schenkelberg, Rosenkranz, Milliken, Menear, & Dzewaltowski, 2017)
- Inadequate tool (n=8): The tool did not provide sufficient information, such as the inadequate detail or lack of reliability/validity (e.g. Boddy, Downs, Knowles, & Fairclough, 2015)
- Confounding variables (n=9): Another unforeseen variable may have affected the outcomes of the research, such as learning effects from testing at multiple times (e.g. Chu & Pan, 2012)
- Limited causality (n=10): Due to the cross-sectional nature of the research, causality cannot be determined (e.g. Memari, Mirfazeli, Kordi, Shayestehfar, Moshayedi, & Mansournia, 2017)
- Sample generalizability (n=12): Participant demographics lacked heterogeneity, including age, sex, ethnicity, ASD severity, etc. (e.g. Hilton, Crouch, & Israel, 2008)
- Missing measures (n=13): In hindsight, the researcher(s) should have collected more data from the participants, such as baseline IQ or BMI (e.g. Bremer, Balogh, & Lloyd, 2015)
- Sample size (n=18): Very few participants with ASD were included in the research (e.g. Alexander, Dummer, Smeltzer, & Denton, 2011)
